# Pharmacological and Adjunctive Management of Non-Hospitalized COVID-19 Patients During the Omicron Era: A Systematic Review and Meta-Analysis

**DOI:** 10.3390/v17081128

**Published:** 2025-08-16

**Authors:** Lorenzo Vittorio Rindi, Drieda Zaçe, Loredana Sarmati, Roberto Parrella, Gianluca Russo, Massimo Andreoni, Claudio Maria Mastroianni

**Affiliations:** 1 Department of Systems Medicine, Infectious Diseases Clinic, University Hospital “Tor Vergata”, 00133 Rome, Italy; l.rindi@gmail.com (L.V.R.); driedazace@gmail.com (D.Z.); sarmati@med.uniroma2.it (L.S.); andreoni@uniroma2.it (M.A.); 2 Respiratory Infectious Diseases Unit, Cotugno Hospital, AORN dei Colli, 80131 Naples, Italy; rob.parrella@gmail.com; 3 Department of Public Health and Infectious Diseases AOU Policlinico Umberto I Sapienza, 00161 Rome, Italy; gianluca.russo@uniroma1.it

**Keywords:** COVID-19, SARS-CoV-2, systematic review, meta-analysis, remdesivir, nirmatrelvir/ritonavir, antiviral, monoclonal antibodies, sotrovimab, molnupiravir

## Abstract

Introduction: The emergence of SARS-CoV-2 Omicron subvariants characterized by increased transmissibility and immune escape has raised concerns about the efficacy of current treatments. This systematic review and meta-analysis evaluated pharmacological and non-pharmacological interventions in Omicron-infected non-hospitalized patients, focusing on key clinical outcomes such as hospitalization, respiratory failure, ICU admission, and 30-day mortality. Methods: Searches were performed in MEDLINE, EMBASE, Web of Science, Cochrane, and ClinicalTrials.gov (last update: 13 July 2025). Eligible studies reported outcomes on antiviral agents, monoclonal antibodies, adjunctive therapies, or telemedicine. Random-effects meta-analyses were conducted when appropriate, with heterogeneity assessed by I^2^. Publication bias was evaluated via funnel plots and Egger’s test. Subgroup analyses explored sources of heterogeneity. Results: Eighty-eight studies were included. Meta-analyses, comparing treatment vs. no treatment, revealed that nirmatrelvir/ritonavir reduced hospitalization by 52% (RR 0.48, 95% CI 0.36–0.63) and all-cause mortality by 84% (RR 0.16, 95% CI 0.11–0.24). Remdesivir reduced hospitalization by 70% (RR 0.30, 95% CI 0.19–0.47) and respiratory failure by 89% (RR 0.11, 95% CI 0.03–0.44). Sotrovimab decreased hospitalization (RR 0.71, 95% CI 0.54–0.93) and mortality (RR 0.34, 95% CI 0.19–0.61). Molnupiravir modestly reduced hospitalization (RR 0.80, 95% CI 0.70–0.91) and respiratory failure (RR 0.45, 95% CI 0.27–0.77). Conclusions: Nirmatrelvir/ritonavir and remdesivir remain important for reducing severe outcomes, while sotrovimab retains partial efficacy. Rapid access to antivirals remains an important factor in mitigating SARS-CoV-2’s burden.

## 1. Introduction

The COVID-19 pandemic, driven by the severe acute respiratory syndrome coronavirus 2 (SARS-CoV-2), has presented unprecedented challenges to global healthcare systems since its emergence in late 2019 [1].

After circulating in humans, SARS-CoV-2 underwent significant adaptations, leading to highly mutated forms known as ‘variants of concern’ (VOCs), classified as Alpha, Beta, Gamma, Delta, and Omicron, emerging independently and quickly became dominant due to their enhanced viral fitness, transmission capabilities, and immune escape compared to previous variants [2].

Since its initial identification in November 2021, the Omicron lineage (particularly BA.1.1 and BA.2) rapidly became the dominant variant globally by early 2022, followed by the emergence of multiple sublineages such as XBB.1.5, which predominated by early 2023 [3,4]. As of August 2025, NB.1.8.1 and LP.8.1 subvariants accounted for approximately 74% of new infections in the United States [4]. Despite increased infection rates, these recent variants did not result in proportional rises in hospitalizations and mortality [5,6]. This evolving epidemiological landscape highlights the need for updated clinical management and treatment strategies.

This systematic review and meta-analysis aimed to investigate key aspects related to SARS-CoV-2 non-hospitalized patient management, limiting our inclusion to data relevant to the viral Omicron lineages prevalent after 1 January 2022. We evaluated treatment strategies including antiviral therapy, monoclonal antibodies, and non-antiviral adjunctive treatments, assessing their roles in preventing hospitalization, mortality, respiratory failure, and disease progression. Additionally, we examined recent studies in the literature for management strategies such as telemedicine, assessing its impact on clinical outcomes, patient satisfaction, and healthcare accessibility. By focusing on these aspects, we aimed to provide a comprehensive overview of effective treatment and management strategies for SARS-CoV-2 non-hospitalized patients in the context of the evolving pandemic.

## 2. Methods

This systematic review adheres to the Preferred Reporting Items for Systematic Reviews and Meta-Analyses (PRISMA) [7] guidelines and is based on a standardized pre-planned analysis available on PROSPERO (CRD42024538332).

## 3. Search Question

The present work aims to answer the following search question: “In the adult population of non-hospitalized patients with SARS-COV-2 infection since the rise of omicron variants, do management strategies such as early pharmacological treatment and telemedicine, represent a benefit in terms of preventing hospitalization, mortality, disease progression or respiratory failure?”

The research question was structured based on the PI/ECOS (T) statement as follows:

- Population: adult population with recent diagnosis (within 7 days) of SARS-CoV-2 infection since the rise in Omicron variants.
- Intervention: management strategies in SARS-CoV-2-infected non-hospitalized patients, including pharmacological treatment and telemedicine.
- Comparison: no such strategies or any other pharmacological treatment against SARS-CoV-2 infection.
- Outcome: hospitalization, mortality, disease progression (Intensive Care Unit (ICU) admission), and respiratory failure.
- Setting: non-hospitalized patients.
- Study design: RCT, observational studies, and NRIS.
- Time period: since 1 January 2022.

## 4. Search and Selection Process

The electronic databases of MEDLINE, Web of Science, EMBASE, Cochrane, and ClinicalTrials.gov were searched on 25 April 2024. A search string for PubMed, consisting of Medical Subject Headings terms and free text words, was developed (Supplementary Material S1). An update of the search strategy was performed on 13 July 2025.

Results were merged in the computerized database, Rayyan, for deduplication and screening by title and abstract [8]. Screening was performed blinded by LVR and DZ. Conflicts were solved by consensus or by consulting a third reviewer. 

After screening by title and abstract, remaining records were assessed for inclusion by reading the study’s full text, obtaining an inclusion list of records proceeding to the extraction phase, performed blinded by LVZ and DZ. Conflicts were solved by consensus. The following data were extracted by two independent reviewers (DZ and LVR): study details (authors, publication year, study design, country, study period, and length of follow-up), participants’ information (number of participants, study population, age, and gender), intervention details (pharmacological or non-pharmacological interventions), and outcome (hospitalization rate and predicting factors, ICU admission, need for oxygen support, death rate and predicting factors, adverse events, rate and time of viral clearance, time to alleviation of symptoms, post-COVID-19 syndrome, and patients’ satisfaction).

## 5. Inclusion and Exclusion Criteria

We limited our search to fully published records reporting the outcome of any pharmacological or non-pharmacological interventions in non-hospitalized patients with ongoing SARS-CoV-2 infection. Outcomes of interest included hospitalization rate and predicting factors, ICU admission, need for oxygen support, death rate and predicting factors, adverse events, rate and time of viral clearance, time to alleviation of symptoms, post-COVID-19 syndrome, and patients’ satisfaction. We excluded any modeling studies, simulation trials, studies that referred to ambulatory management of other pathologies during the COVID-19 pandemic, and studies that reported data referring to both the Omicron era and before, without the possibility to discriminate between them. Case reports/case series, conference abstracts, conference papers, reviews, meta-analyses, editorials, and commentaries were also excluded. No further restrictions in terms of country, setting, or language were made.

## 6. Data Analysis

Data was grouped according to the type of intervention implemented and divided into pharmacological (therapies targeting SARS-CoV-2; therapies not targeting SARS-CoV-2; adjuvant therapies) or non-pharmacological interventions (telemedicine; organizational pathways; follow-up). For the studies reporting the same outcome for the same intervention, we performed random-effect meta-analyses, reporting pooled data with 95% confidence intervals (CIs). Heterogeneity was measured by I^2^ statistics. Forest plots were used for the graphical representation of each meta-analysis. Publication bias was examined graphically through funnel plots and Egger’s test, where *p* < 0.05 indicates significant publication bias [9]. Subgroup analyses were performed to explore potential sources of variability among the meta-analyses with high heterogeneity. The key potential sources of heterogeneity considered were study design (retrospective vs. prospective), period of data collection (studies with data collected during 2022), sample size (studies with more than 5 events in each group), and population (general vs. particular groups). Furthermore, given the high degree of heterogeneity even after the subgroup analyses, we ran a meta-regression to regress the RR upon studies’ sample size, age categories (studies with median ages > 60 years old), and vaccination status (≤50% vs. >50% of the population vaccinated). The meta-analyses were performed on Stata v.15.0 software (Stata Corp, College Station, TX, USA). 

## 7. Quality Appraisal of Included Studies

Quality assessment was performed for all included studies, blinded, by LVZ and DZ. Conflicts were solved by consensus. Randomized studies were assessed by the Cochrane Risk of Bias tool v.2 [10]; non-randomized interventional studies were evaluated by the ROBINS-I tool [11], while observational studies were assessed by the Newcastle–Ottawa Scale (NOS) [12].

The evidence deriving from the systematic review and meta-analysis was presented according to the GRADE framework in order to rank its certainty (Supplementary Material S4).

## 8. Results

**Figure 1:**
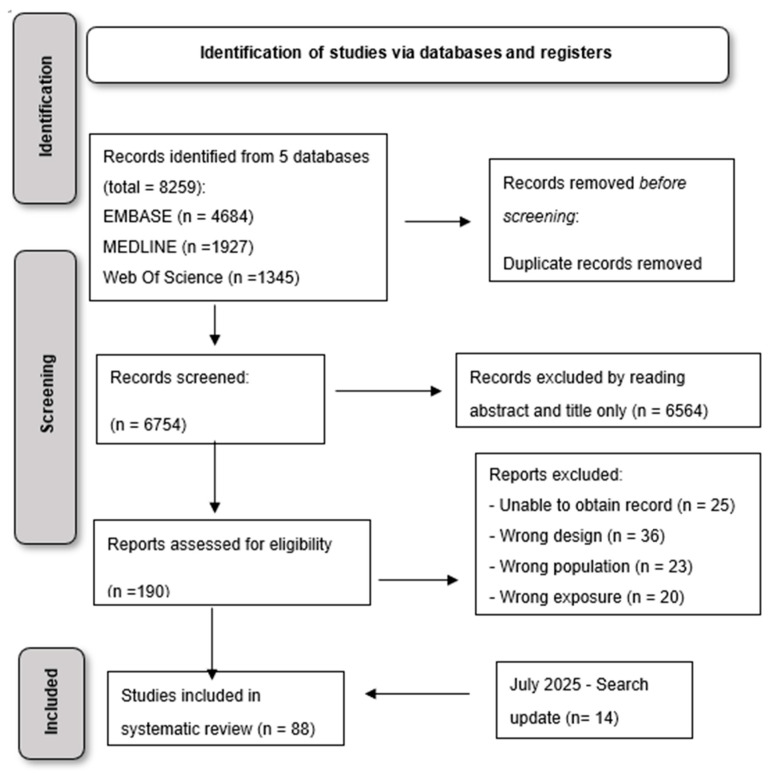
PRISMA flowchart of the included studies.

Our search strategy produced a total number of 8259 records. After duplicate removal, a total of 6754 records were screened by title/abstract and assessed for inclusion criteria. Figure 1 presents the results of the screening and selection process, including a search update which was rerun prior to submission, identifying a total of 1148 new articles. At the end of the screening process, 14 new articles (3 RCTs and 11 observational studies) were added to the systematic review, for a total of 88 records.

## 9. Risk of Bias

Out of the 16 randomized studies assessed with the RoB2 tool, 1 was determined to have some bias, 1 was categorized as having a high risk of bias, and the remaining studies were found to have a low risk of bias. Among the observational studies assessed with the NOS, 46% (33/72) met all nine quality criteria (9/9), 31% (22/72) fulfilled eight out of nine criteria (8/9), and 18% (13/72) met seven out of nine criteria (7/9). A detailed analysis can be found in Supplementary Material S2.

## 10. Pharmacological Interventions

### Antivirals

Sixty-three eligible articles investigated the role of antivirals in the early management of SARS-CoV-2 infection during the Omicron era [13,14,15,16,17,18,19,20,21,22,23,24,25,26,27,28,29,30,31,32,33,34,35,36,37,38,39,40,41,42,43,44,45,46,47,48,49,50,51,52,53,54,55,56,57,58,59,60,61,62,63,64,65,66,67,68,69,70,71,72,73,74,75]. Specifically, 34 studies included treatment arms including molnupiravir, 38 examined nirmatrelvir/ritonavir, and 17 assessed remdesivir use, while other strategies examined the potential use of pomotrelvir, ensiltrevir, and favipiravir [13,14,15,16,17,18,19,20,21,22,23,24,25,26,27,28,29,30,31,32,33,34,35,36,37,38,39,40,41,42,43,44,45,46,47,48,49,50,51,52,53,54,55,56,57,58,59,60,61,62,63,64]. Favipiravir was examined by an RCT and compared to molnupiravir, showing a similar safety and efficacy profile [36]. Pomotrelvir was not found to be superior to placebo in the single study eligible for inclusion [18].

Studies discussing molnupiravir, remdesivir, and nirmatrelvir/ritonavir reported sufficient data for separate meta-analyses for each of the included outcomes.

## 11. Remdesivir

**Figure 2:**
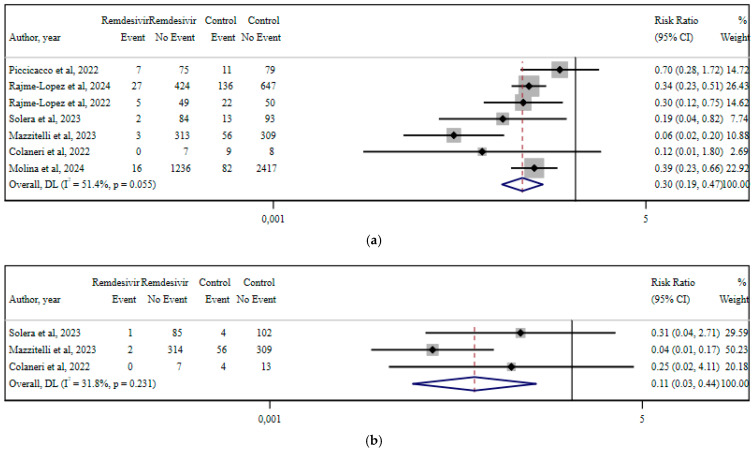

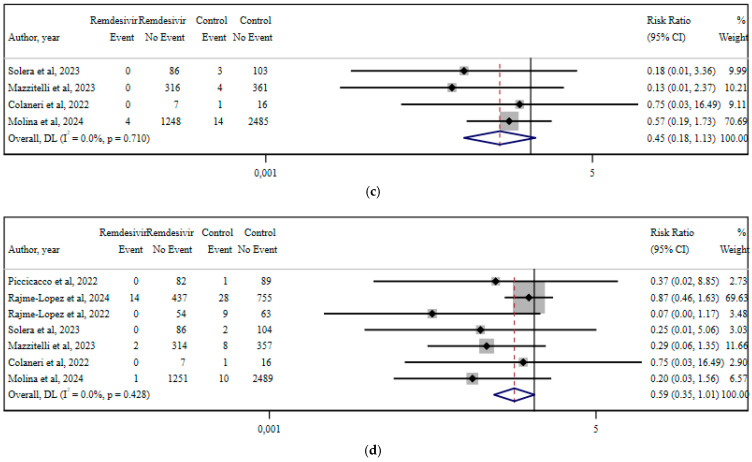
Forest plots of included meta-analyses for each of the investigated outcomes in early remdesivir treatment vs. no intervention. Results are depicted as RR for the reported outcomes: (**a**) hospitalization; (**b**) respiratory failure; (**c**) ICU admission; and (**d**) all-cause mortality [16,24,34,52,60,61,67].

Seven studies reporting data about early remdesivir use vs. no therapy in non-hospitalized COVID-19 patients contained sufficient data to perform separate meta-analyses for each of the investigated outcomes, i.e., hospitalization risk, respiratory failure, ICU admittance, and all-cause 30-day mortality (Figure 2) [16,24,34,52,60,61,67].

When comparing the RR of hospitalization in the case of early remdesivir administration vs. no treatment, remdesivir reduced hospitalization by 70%, RR 0.30 (95% CI, 0.19–0.47; I^2^ 51.4%, *p* = 0.055). Respiratory failure was reduced by 89%, RR 0.11 (95% CI, 0.03–0.44; I^2^ 31.8%, *p* = 0.231). RR for ICU admittance was calculated to be 0.45 (95% CI, 0.18–1.13; I^2^ 0%, *p* = 0.71), not reaching statistical significance. Finally, 30-day all-cause mortality was reduced by 41% in the treated cohorts of meta-analyzed studies (RR 0.59, 95% CI, 0.35–1.01; I^2^ 0%, *p* = 0.428), although it was not statistically significant [16,24,34,52,60,61,67]. The grading of evidence documented a low or very low certainty of evidence for the studies that could be meta-analyzed, mainly because of study design (observational studies) and publication bias. 

## 12. Nirmatrelvir/Ritonavir

**Figure 3:**
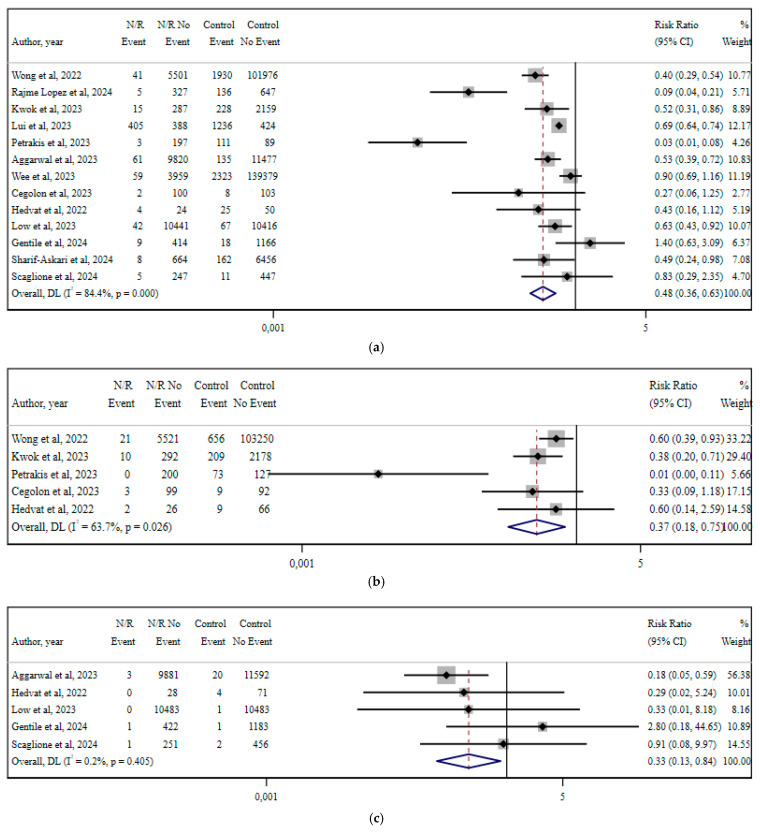

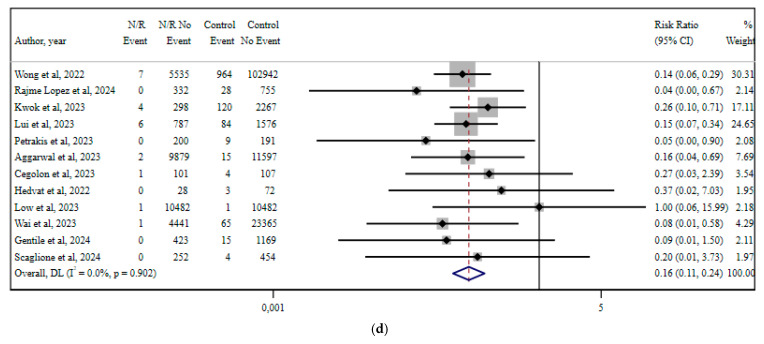
Meta-analyses for nirmatrelvir/ritonavir administration vs. no treatment. Results are depicted as RR for the reported outcomes: (**a**) hospitalization; (**b**) respiratory failure; (**c**) ICU admission; and (**d**) all-cause mortality [14,26,30,31,32,39,42,45,47,55,60,65,70,74].

Fourteen studies reported sufficient data for meta-analyzing the effect of early administration of nirmatrelvir/ritonavir (vs. no treatment) on hospitalization risk, onset of respiratory failure, ICU admission, and all-cause mortality [14,26,30,31,32,39,42,45,47,55,60,65,70,74] (Figure 3). Early nirmatrelvir/ritonavir treatment reduced hospitalization risk by 52% (RR 0.48; 95% CI 0.36–0.63; I^2^ 84.4%; *p* < 0.001) and was associated with a 63% lower likelihood of respiratory failure (RR 0.37; 95% CI 0.18–0.75; I^2^ 63.7%; *p* = 0.026). The RR for ICU admission was 0.33 (95% CI 0.13–0.84; I^2^ 0.2%; *p* = 0.405), while all-cause mortality was reduced by 84% (RR 0.16; 95% CI 0.11–0.24; I^2^ 0%; *p* = 0.902) [14,26,30,31,32,39,42,45,47,55,60].

The subgroup analysis, stratified by study design, data collection period, population characteristics, and sample size, revealed that the greatest reduction in heterogeneity was observed when including only studies that collected data in 2022. In this subgroup, heterogeneity decreased, with an I^2^ value of 64%, suggesting that temporal factors related to the data collection period may have contributed to the variability observed in the overall analysis (Supplementary Material S3). The meta regression included, as variables, the sample size of the studies, age, and the vaccination status of participants at the study level, as well as the reported I^2^_res_ statistic of 10.4% and the adjusted R^2^ statistic of 100%. Results showed that larger sample sizes were significantly associated with a greater reduction in the relative risk, while higher vaccination coverage was significantly associated with a smaller relative risk reduction (Table 1).

Publication bias

**Figure 4:**
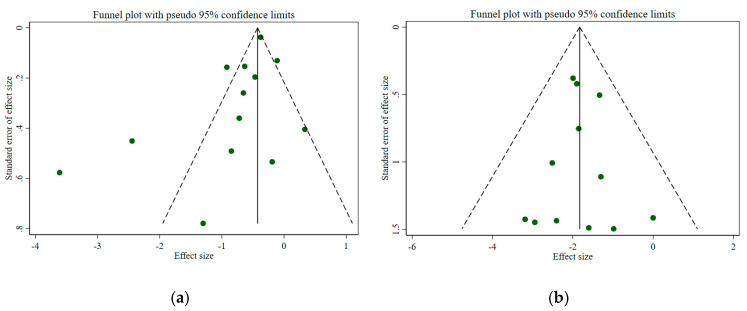
Funnel plots for NR reporting the outcomes of (**a**) hospitalization and (**b**) mortality.

For the meta-analysis evaluating the outcomes of hospitalization and mortality associated with N/R, publication bias was assessed using funnel plots and Egger’s test, given that more than 10 studies were included (Figure 4). Evidence of potential publication bias was observed among studies reporting hospitalization (Egger’s test *p* = 0.03), whereas no significant publication bias was detected for studies reporting *mortality* (Egger’s test *p* = 0.24).

The grading of evidence using GRADE-pro documented a low or very low certainty of evidence for the studies that could be meta-analyzed, mainly because of study design (observational studies) and publication bias.

## 13. Molnupiravir

**Figure 5:**
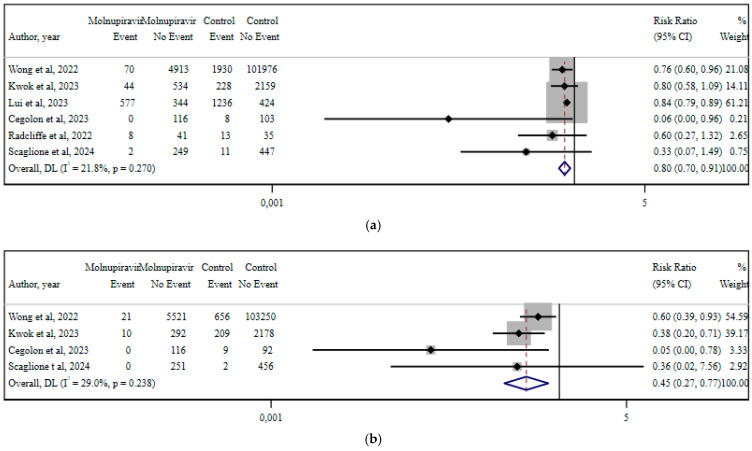

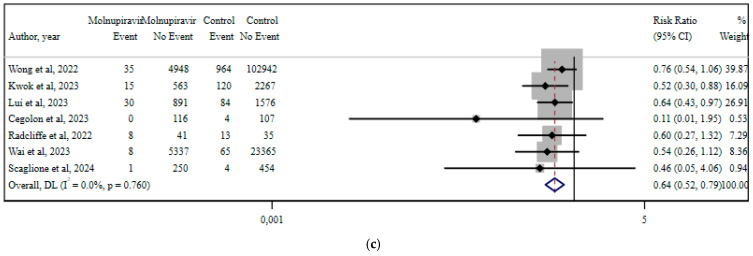
Meta-analysis for molnupiravir use vs. no intervention. Results are depicted as RR for the reported outcomes: (**a**) hospitalization; (**b**) respiratory failure; and (**c**) 30-day all-cause mortality [14,26,30,42,45,48,74].

Figure 5 summarizes studies comparing early molnupiravir therapy to no treatment in non-hospitalized COVID-19 patients included in the meta-analyses evaluating hospitalization risk, respiratory failure, and 30-day all-cause mortality [14,26,30,42,45,48,74]. With early treatment with molnupiravir, hospitalization was less likely by 20% (RR 0.80; 95% CI 0.70–0.91; I^2^ %; *p* = 0.270), while respiratory failure risk was reduced by 55% (RR 0.45; 95% CI 0.27–0.77; I^2^ 29%; *p* = 0.238). All-cause mortality at 30 days was 36% less likely (RR 0.64; 95% CI 0.52–0.79; I^2^ 0%; *p* = 0.76) [14,26,30,42,45,48].

The grading of evidence showed a low or very low certainty of evidence, mainly because of study design (observational studies) and publication bias.

### 13.1. Monoclonal Antibodies

Nineteen studies assessed the role of monoclonal antibodies in preventing disease progression in SARS-CoV-2 infection [16,17,21,28,42,43,44,47,48,62,75,76,77,78,79,80,81,82,83]. Of these, fourteen focused on sotrovimab and bebtelovimab, and two focused on casirivimab/imdevimab or bamlanivimab/etesevimab. The latter two regimens demonstrated limited efficacy compared to sotrovimab [80,81].

Treatment with bebtelovimab was associated with reduced hospitalization rates, an effect that was particularly pronounced in patients with two or more comorbidities [78,82,83]. Among the included studies, data were sufficient to conduct separate meta-analyses for sotrovimab only.

### 13.2. Sotrovimab

**Figure 6:**
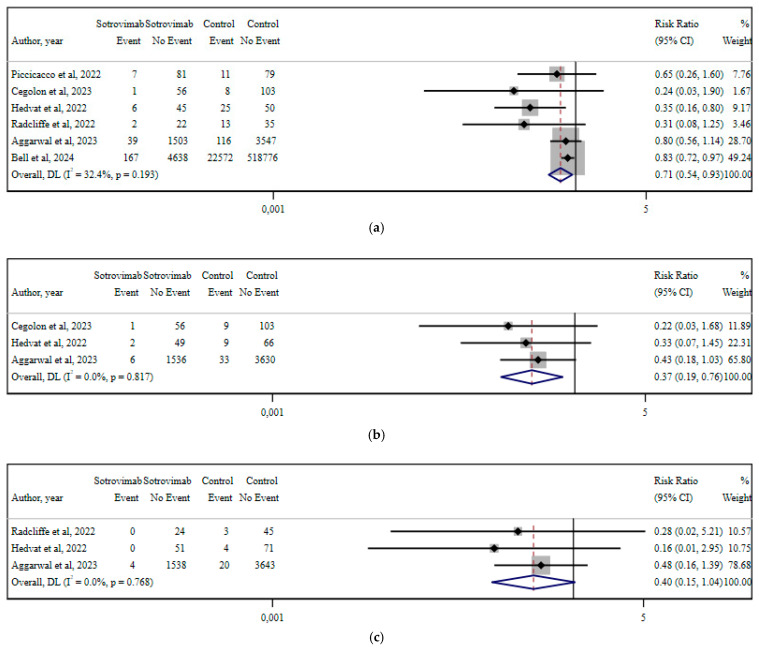

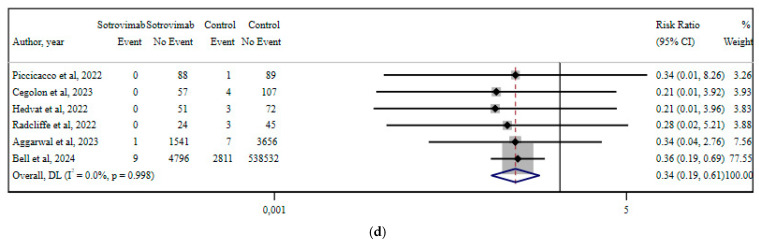
Forest plots of included meta-analyses for each of the investigated outcomes in early sotrovimab treatment vs. no intervention. Results are depicted as RR for the reported outcomes: (**a**) hospitalization; (**b**) respiratory failure; (**c**) ICU admission; and (**d**) 30-day all-cause mortality [16,42,47,48,77,79].

Meta-analyses of six studies compared the outcomes of early sotrovimab use versus no treatment in non-hospitalized COVID-19 patients [16,42,47,48,77,79] (Figure 6). The analyzed outcomes included hospitalization risk, respiratory failure, ICU admission, and 30-day all-cause mortality. Early sotrovimab therapy was associated with a 29% reduction in hospitalization risk (RR 0.71; 95% CI 0.54–0.93; I^2^ 32.4%; *p* = 0.193) [16,42,47,77,79]. The risk of respiratory failure was reduced by 63% (RR 0.37; 95% CI 0.19–0.76; I^2^ 0%; *p* = 0.817) [42,47,77]. ICU admission showed an RR of 0.40 (95% CI 0.15–1.04; I^2^ 0%; *p* = 0.768) [47,48,77], with no statistical significance. Lastly, 30-day all-cause mortality was 66% lower in patients receiving sotrovimab (RR 0.34; 95% CI 0.19–0.61; I^2^ 0%; *p* = 0.998) [16,42,47,48,77,79].

The grading of evidence reported a low or very low certainty of evidence, mainly because of study design (observational studies) and publication bias.

### 13.3. Adjuvant Therapy

Eight eligible studies explored a range of medications for potential efficacy against COVID-19. Among these, two evaluated fluvoxamine, while single studies investigated bromhexine, domperidone, inhaled IBIO123, bosentan, probenecid, and halofuginone. However, none of these treatments demonstrated consistent or significant benefits in reducing hospitalization rates or preventing disease progression [84,85,86,87,88,89,90,91].

### 13.4. Telemedicine

Four studies assessed the role of telemedicine in managing COVID-19 outpatients. Vargas et al. reported that telephonic follow-up during the fourth pandemic wave (January–April 2022) in primary care settings reduced the risk of all-cause mortality among ambulatory patients [92]. Mandal et al. analyzed video-based telemedicine usage in New York City for a total of 2,748,635 televisits and remote urgent care services (Virtual Urgent Care—13,669 visits) during the pandemic, particularly during the Omicron wave (November 2021–January 2022). Researchers report its usefulness in both the management of COVID-19 (suspect/confirmed) and in urgent care, with great patient satisfaction, especially by younger age groups, and with a positive effect on care accessibility and equity [93].

Two studies explored telemedicine’s role in kidney transplant recipients. Liew et al. followed 81 kidney transplant patients with COVID-19, who were managed via teleconsultation and a hospital-at-home model between February and June 2022. Of these, 86.4% recovered without complications, while 13.6% required hospitalization [94].

Zahradka et al. demonstrated that a structured telemedicine program improved communication, diagnostics, and treatment for kidney transplant patients with COVID-19, emphasizing its broader applicability to vulnerable populations [95].

## 14. Organizational Pathways and Follow-Up

Timely intervention and follow-up are essential for preventing disease progression in non-hospitalized COVID-19 patients. A study on 262 patients described a clinical pathway involving detection and referral systems, day-hospital evaluation, and subsequent follow-up, ensuring equitable access to treatment and improved outcomes [96].

Three studies highlighted follow-up strategies. One study involving 706,412 confirmed COVID-19 cases during the fourth wave found that telephonic follow-up reduced mortality risk in outpatients with mild COVID-19 [92].

## 15. Discussion

This review and meta-analysis aimed at exploring the role of early pharmacological and non-pharmacological interventions in the outpatient management of COVID-19 during the Omicron era. Based on the identified studies, we could provide meta-analytical data only about the efficaciousness of the currently available therapies (antivirals and monoclonal antibodies) for the most important outcomes, i.e., risk of hospitalization, respiratory failure, ICU admission, and all-cause mortality, comparing their risk to those of control groups receiving no treatment. For non-pharmacological interventions, a descriptive synthesis was conducted. In order to provide an up-to-date review, we limited our inclusion timespan to the period of exclusive dominance of the VOC Omicron, excluding evidence from studies including previous VOCs. Each treatment option, including remdesivir, nirmatrelvir/ritonavir, molnupiravir, and sotrovimab, was associated with reductions in the studied outcomes, although the significance and magnitude of these effects varied among therapies.

Antiviral therapies such as remdesivir, nirmatrelvir/ritonavir, and molnupiravir demonstrated high efficacy in reducing hospitalization, respiratory failure, and mortality, compared to no treatment. Specifically, remdesivir showed a significant reduction in hospitalization and respiratory failure, while for ICU admission and mortality, there was a favorable trend, although the wide confidence interval and lack of statistical significance suggest insufficient evidence.

Notably, nirmatrelvir/ritonavir showed consistent and robust reductions in all key outcomes, reflecting its potential as a cornerstone of outpatient therapy. The data concerning this antiviral reached statistical significance in reducing hospitalization, respiratory failure, ICU admission, and mortality. However, the high heterogeneity for hospitalization and respiratory failure warrants caution, likely reflecting variability in study designs or patient populations. To explore the sources of heterogeneity, subgroup analyses were conducted. Among the variables examined, the data collection period appeared to have the greatest impact on heterogeneity, even though it still remained moderate (I^2^ 64%). Hence, a meta-regression was run, which showed that larger sample sizes were significantly associated with a greater reduction in the relative risk, suggesting that small studies may have reported less pronounced treatment effects and higher vaccination coverage was significantly associated with a smaller relative risk reduction, indicating that the apparent benefit of treatment was less pronounced in populations with a greater vaccination rate. Both of these study-level covariates explained almost all of the heterogeneity.

On the other hand, early treatment with molnupiravir showed more modest benefits. It reduced the risk of hospitalization, respiratory failure, and mortality at 30 days, all being statistically significant results with a low level of observed heterogeneity. These results suggest that molnupiravir may be less effective than nirmatrelvir/ritonavir or remdesivir in achieving these outcomes; however, it remains a viable option, particularly for patients who cannot receive other antivirals due to issues such as drug–drug interactions or renal impairment.

Such results, including findings about remdesivir use and the modest benefit of molnupiravir compared to its counterparts, are sustained by the current literature [97,98,99,100].

As for monoclonal antibodies, sotrovimab demonstrated a reduction in the risk of hospitalization, respiratory failure, and mortality, while for ICU admission, statistical significance was not reached.

Treatment choice among these drugs should be considered on a personalized basis, including a consideration of patient-specific risk factors, settings, drug–drug interactions, drug availability, and infrastructural issues, e.g., organizational and structural restrictions to the possibility of outpatient administration of 3-day remdesivir. The role of monoclonal antibodies, particularly sotrovimab, remains an option, albeit with diminishing efficacy against newer variants. Earlier combinations such as casirivimab/imdevimab exhibited markedly reduced effectiveness during the Omicron surge [101].

While repurposed medications may provide theoretical benefits, the lack of consistent clinical efficacy in reducing disease progression underscores the importance of rigorous investigation before widespread adoption.

The results of this study should be interpreted in the light of some limitations, including the heterogeneity of study designs, differences in patient populations, and the reliance on observational data for many analyses. The generalizability of these results to new variants and healthcare settings with differing infrastructure could be uncertain. Furthermore, due to the lack of data, we were unable to perform meta-analyses to directly compare the different treatments with one another, limiting our analysis to comparisons with the no-treatment group. Furthermore, GRADE evaluation showed that all evidence included in the meta-analyses had a very low or low certainty of evidence. As a result, no conclusions can be drawn regarding the relative efficacy of these therapies. Future research should focus on direct comparisons of these therapies and their effectiveness in specific subgroups, such as immunocompromised patients or those with comorbidities. Additionally, high heterogeneity in certain endpoints, such as hospitalization, underscores the need for further investigation into population-specific responses and potential confounding factors.

Telemedicine may play an important role, particularly among vulnerable populations, such as immunocompromised individuals, where structured telemedicine programs can provide critical support. Telephonic follow-up, in particular, demonstrated an association with reduced mortality, proving its value as a scalable and cost-effective intervention. Similarly, video-based telemedicine facilitated patient adaptability for both routine and urgent care needs [93,95]. However, the effectiveness and implementation of telemedicine strategies may vary significantly depending on the epidemiological context, healthcare infrastructure, and population needs. Despite these promising findings, the evidence base remains limited. Only a small number of observational studies specifically assessed telemedicine interventions, and many lacked quantitative outcome measures, precluding formal meta-analysis. Furthermore, heterogeneity in study designs, populations, and intervention modalities limits the generalizability of the conclusions.

Future research should aim to evaluate the long-term effectiveness, equity, and cost-effectiveness of telemedicine programs, with particular attention to their integration into routine clinical pathways for the management of chronic conditions and support of high-risk groups. Well-designed prospective studies and randomized controlled trials are needed to provide more robust evidence and guide policy development.

## 16. Conclusions

In conclusion, this systematic review highlights the potential of early pharmacological interventions in optimizing the management of non-hospitalized COVID-19 patients during the Omicron era. Nirmatrelvir/ritonavir, with its reported reductions in both mortality and severe disease markers, appears to be an effective option, compared to no treatment, as well as remdesivir and sotrovimab. Molnupiravir, while less effective overall, remains an alternative for patients unable to access other treatments. However, findings are tempered by the heterogeneity of study designs, the observational nature of much of the data, and variability in healthcare settings among included studies. Future research should address gaps in head-to-head comparisons of interventions and the integration of pharmacological and non-pharmacological approaches into standardized care pathways. A multidisciplinary and adaptable approach remains essential to enhance patient outcomes.

## Figures and Tables

**Table 1:** Meta-regression results for the relative risk of hospitalization in patients treated with N/R compared with those receiving no treatment.

Variables	Coefficient (CI, 95%)	Standard Error	*p*-Value
**Sample size**			
≤5 events in each group	REF		
>5 events in each group	−2.1 (−3.4 to −0.7)	0.5	0.01
**Age**			
Median age ≤ 60 years	REF		
Median age > 60 years	0.8 (−0.6 to 2.2)	0.5	0.17
**Vaccination**			
≤50% of the study population vaccinated	REF		
>50% of the study population vaccinated	1.5 (0.2–2.8)	0.4	0.03

## Data Availability

All data produced or analyzed in this study are presented within this published article and its Supplementary Materials.

## Supplementary Materials

The following supporting information can be downloaded at: https://www.mdpi.com/article/10.3390/v17081128/s1, Supplementary Material S1: Search strategy; Supplementary Material S2: Quality assessment of the included studies; Supplementary Material S3: Subgroup analysis of studies included in the meta-analysis of N/R for the outcome “hospitalization”; Supplementary Material S4: Grading of evidence of the studies included in the metanalyses using GRADE-pro.
