# Supplementary material for: Pharmacological and Adjunctive Management of Non-Hospitalized COVID-19 Patients During the Omicron Era: A Systematic Review and Meta-Analysis
Source: Viruses. 2025 Aug 16;17(8):1128. doi: 10.3390/v17081128 (PMC12390715; doi:10.3390/v17081128)
Supplement: Supplementary file 1 [file viruses-17-01128-s001.zip › Supplementary material S1. Search Strategy(1).pdf]

Supplementary material S1. Search strategy

Search was performed on April 25th, 2024

MEDLINE - 1927 records

(("COVID-19"[Mesh] OR "SARS-CoV-2"[Mesh] OR "2019-nCoV Infection" OR "SARS-CoV-2 Infection" OR "2019 Novel Coronavirus Disease" OR "COVID-19 Virus Infection" OR COVID19 OR "Coronavirus Disease 2019" OR "Severe Acute Respiratory Syndrome Coronavirus 2 Infection" OR "SARS Coronavirus 2 Infection") AND ("Outpatients"[Mesh] OR "Out-patient*" OR "ambulatory care" OR ambulator* OR "non hospitalized" OR "non-hospitalized") AND (treatment OR therapy OR therapeutic* OR drug* OR antiviral* OR "monoclonal antibodies" OR management OR "Telemedicine"[Mesh]))

Web Of Science - 1345 records

TS=((("COVID-19" OR "SARS-CoV-2" OR "2019-nCoV Infection" OR "SARS-CoV-2 Infection" OR "2019 Novel Coronavirus Disease" OR "COVID-19 Virus Infection" OR COVID19 OR "Coronavirus Disease 2019" OR "Severe Acute Respiratory Syndrome Coronavirus 2 Infection" OR "SARS Coronavirus 2 Infection") AND ("Outpatients" OR "Out-patient*" OR "ambulatory care" OR ambulator* OR "non hospitalized" OR "non-hospitalized") AND (treatment OR therapy OR therapeutic* OR drug* OR antiviral* OR "monoclonal antibodies" OR management OR "Telemedicine")))

EMBASE - 4684 records

('coronavirus disease 2019'/exp OR 'severe acute respiratory syndrome coronavirus 2'/exp OR '2019-ncov infection' OR 'sars-cov-2 infection' OR '2019 novel coronavirus disease' OR 'covid-19 virus infection' OR 'covid19' OR 'coronavirus disease 2019' OR 'severe acute respiratory syndrome coronavirus 2 infection' OR 'sars coronavirus 2 infection') AND ('outpatient'/exp OR 'out-patient*' OR 'ambulatory care' OR 'ambulator*' OR 'non hospitalized' OR 'non-hospitalized') AND ('management'/exp OR 'antiviral*' OR 'monoclonal antibodies'/exp)

Clinicaltrials.gov - 18 records

Cochrane Library - 285 records

AB=("COVID-19"[Mesh] OR "SARS-CoV-2"[Mesh] OR "2019-nCoV Infection" OR "SARS-CoV-2 Infection" OR "2019 Novel Coronavirus Disease" OR "COVID-19 Virus Infection" OR COVID19 OR "Coronavirus Disease 2019" OR "Severe Acute Respiratory Syndrome Coronavirus 2 Infection" OR "SARS Coronavirus 2 Infection")

AND

("Outpatients"[Mesh] OR "Out-patient*" OR "ambulatory care" OR ambulator* OR "non hospitalized" OR "non-hospitalized")

AND

(treatment OR therapy OR therapeutic* OR drug* OR antiviral* OR "monoclonal antibodies" OR management OR "Telemedicine"[Mesh])
