# Supplementary material for: Pharmacological and Adjunctive Management of Non-Hospitalized COVID-19 Patients During the Omicron Era: A Systematic Review and Meta-Analysis
Source: Viruses. 2025 Aug 16;17(8):1128. doi: 10.3390/v17081128 (PMC12390715; doi:10.3390/v17081128)
Supplement: Supplementary file 1 [file viruses-17-01128-s001.zip › Supplementary material S2. Quality assessment up.pdf]

COHORT: Newcastle Ottawa Scale				
First Autor, Year	Selection	Comparability	Outcome	Overall
González-Gómez et al., 2024	1	1	3	5
Wong et al., 2022	4	2	3	9
Radcliffe et al., 2022	3	2	3	8
Pontolillo et al., 2022	4	2	2	8
Streinu-Cercel et al., 2022	4	2	2	8
Dryden-Peterson et al., 2023	4	2	3	9
Mazzitelli et al., 2023	4	2	3	9
Tiseo et al., 2023	4	2	3	9
Colaneri et al., 2022	3	2	3	8
Kimata et al., 2023	4	2	3	9
Low et al., 2023	4	2	3	9
Hedvat et al., 2022	3	1	3	7
Wai et al., 2023	3	1	3	7
Scotto et al., 2023	4	2	3	9
Mazzotta et al., 2023	4	2	3	9
Cegolon et al., 2023	4	2	3	9
Minoia et al., 2023	3	1	3	7
Prajapati et al., 2023	4	2	3	9
Wee et al., 2023	4	2	3	9
Ioannou et al., 2023	3	2	3	8
Shinozaki et al., 2024	4	2	3	9
Colaneri et al., 2024	4	2	3	9
Hiremath et al., 2023	2	1	3	6
Molina et al., 2023	4	2	3	9
McCreary et al., 2022	4	1	3	8
Gershengorn et al., 2023	3	1	3	7
Bell et al., 2024	3	1	3	7
Bruno et al., 2022	4	2	3	9
Piccicacco et al., 2022	4	2	3	9
Kauer et al., 2023	3	2	3	8
Zamani et al., 2022	4	2	3	9
Evans et al., 2023	4	2	3	9
Park HR et al., 2023	3	2	3	8
Park JJ et al., 2023	4	2	3	9
Czarnecka et al., 2022	4	2	3	9
Ramos-Rincón et al., 2023	3	2	3	8
Levy et al., 2024	3	1	3	7
Georgakopoulou et al., 2024	4	2	3	9
Yang et al., 2024	3	1	3	7
Rajme-López et al., 2024	4	2	3	9
Rajme-López et al., 2022	4	1	3	8
Chesdachai S et al., 2024	4	2	3	9
Solera et al., 2023	4	2	3	9
Gaspar et al., 2023	3	2	3	8
Poznański et al., 2022	3	2	3	8
Kwok et al., 2023	3	2	3	8
Cowman et al., 2023	4	2	3	9
Manciulli et al., 2023	3	2	3	8
Lui et al., 2023	4	1	3	8

Rinaldi et al., 2023	4	2	3	9
Petrakis et al., 2023	3	2	3	8
Aggarwal et al., 2023	4	2	3	9
Aggarwal et al., 2023 bis	3	2	3	8
Sanchez et al., 2024	4	2	3	9
Vargas-Sanchez et al., 2023	2	2	3	7
Mandal et al., 2022	3	2	2	7
Liew et al., 2023	3	1	3	7
Zahradka et al., 2023	3	1	3	7
Pinargote-Celorio et al., 2023	3	2	3	8
Edelstein et al., 2023	3	2	3	8
Gentile et al., 2024	4	1	3	8
Hsu et al., 2025	4	2	3	9
Molina et al., 2024	4	2	3	9
Jorda et al., 2025	4	2	3	9
Larsen et al., 2025	3	1	3	7
Sharif-Askari et al., 2024	4	2	2	8
Butt et al., 2024	4	2	2	8
Takazono et al., 2024	3	1	3	7
Bhargava et al., 2024	2	1	2	5
Scaglione et al., 2024	4	2	3	9
Rowan et al., 2025	4	2	3	9
Shaver et al., 2022	NA	NA	NA	NA

	RIS: RoB2					
First Autor, Year	D1	D2	D3	D4	D5	OVERALL
Salvadori et al., 2024	L	L	L	L	L	L
Tomazini et al., 2024	L	L	L	L	L	L
Martin et al., 2023	L	L	L	L	L	L
Shahbazi et al., 2023	L	L	L	L	L	L
Vila Mendez et al., 2023	L	L	L	L	L	L
Farahani et al., 2023	H	H	L	H	L	H
Reis et al., 2023	L	L	L	L	L	L
Rabanal Basalo et al., 2023	L	L	L	L	L	L
Mazzaferri et al., 2022	L	L	L	L	L	L
Borroto-Esoda et al., 2024	L	L	L	L	L	L
Standing et al., 2023	L	L	L	L	L	L
Maranda et al., 2024	L	L	L	L	L	L
Meiser et al., 2024	L	L	L	L	L	L
Wei et al., 2025	L	L	L	L	L	L
Jittamala et al., 2024	L	SC	L	SC	L	SC
Siripongboonsitti et al., 2023	L	L	L	L	L	L
