# Supplementary material for: Pharmacological and Adjunctive Management of Non-Hospitalized COVID-19 Patients During the Omicron Era: A Systematic Review and Meta-Analysis
Source: Viruses. 2025 Aug 16;17(8):1128. doi: 10.3390/v17081128 (PMC12390715; doi:10.3390/v17081128)
Supplement: Supplementary file 1 [file viruses-17-01128-s001.zip › Supplementary material S3. Sub group analysis.pdf]

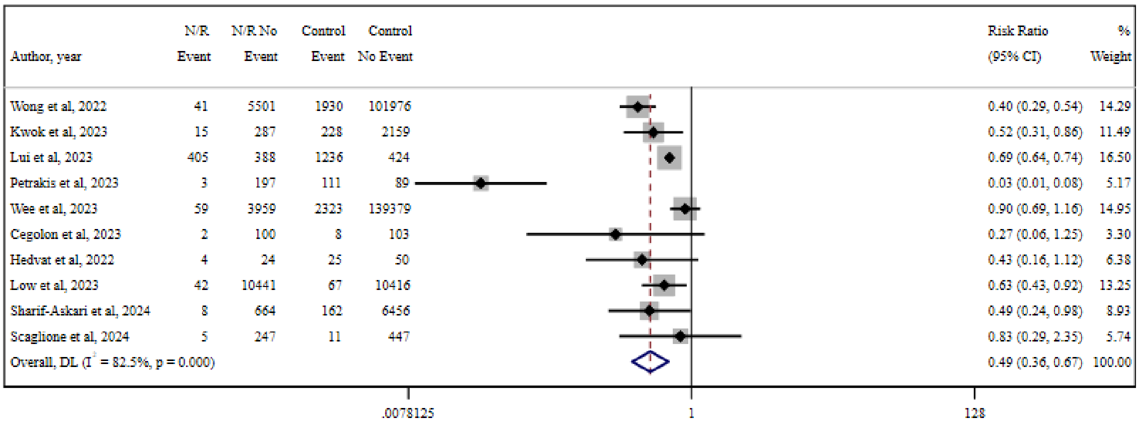

Figure S1 Subgroup analysis of studies included in the meta-analysis of N/R for the outcome “hospitalization,” stratified by study design, including only retrospective studies.

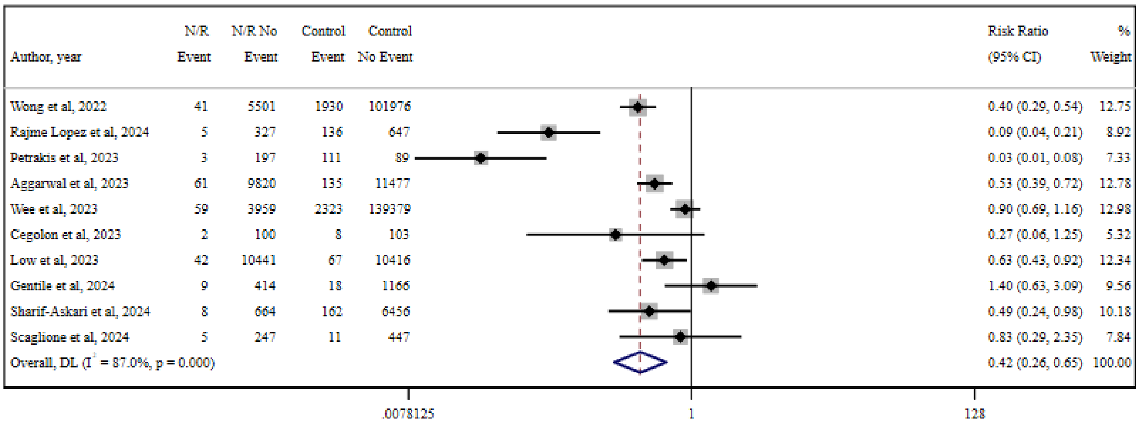

Figure S2 Subgroup analysis of studies included in the meta-analysis of N/R for the outcome “hospitalization,” stratified by population type, including only studies conducted in the general population

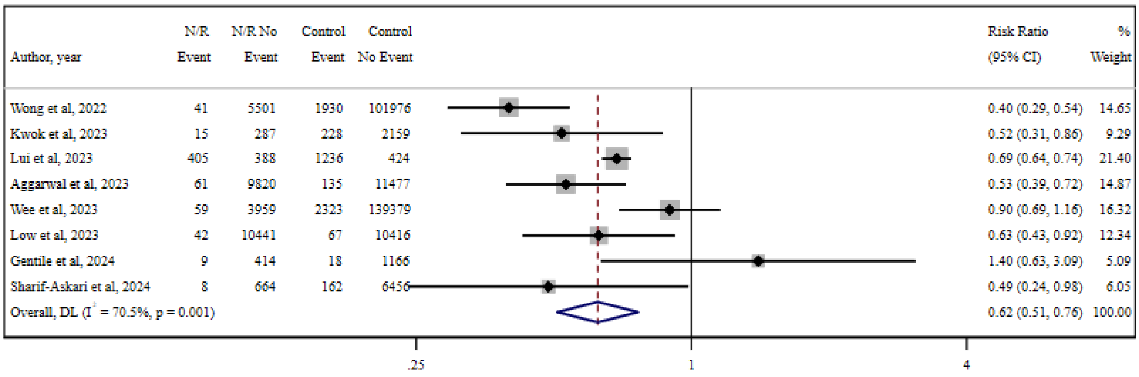

Figure S3 Subgroup analysis of studies included in the meta-analysis of N/R for the outcome “hospitalization,” stratified by sample size, including only studies with >5 events in the treatment and control groups

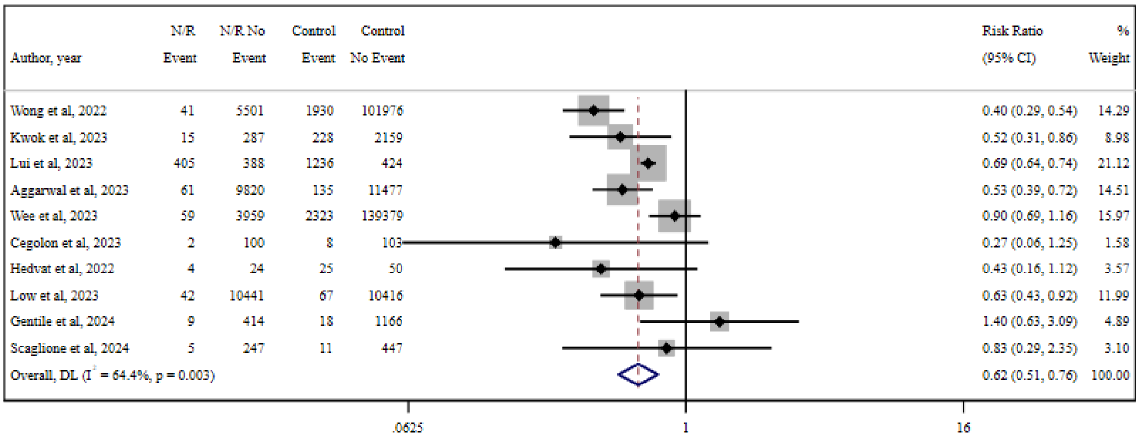

Figure S4 Subgroup analysis of studies included in the meta-analysis of N/R for the outcome “hospitalization,” stratified by sample size, including only studies with data collection conducted during 2022
