# Supplementary material for: Pharmacological and Adjunctive Management of Non-Hospitalized COVID-19 Patients During the Omicron Era: A Systematic Review and Meta-Analysis
Source: Viruses. 2025 Aug 16;17(8):1128. doi: 10.3390/v17081128 (PMC12390715; doi:10.3390/v17081128)
Supplement: Supplementary file 1 [file viruses-17-01128-s001.zip › Supplementary material S4a.GRADE N_R vs no treatment.pdf]

Author(s):
Question: N/R compared to no therapy for hospitalization/respiratory failure/ICU/Mortality reduction
Setting:
Bibliography:

Certainty assessment							№ of patients		Effect		Certainty	Importance
№ of studies	Study design	Risk of bias	Inconsistency	Indirectness	Imprecision	Other considerations	N/R	no therapy	Relative (95% CI)	Absolute (95% CI)		
**Hospitalization**												
13	non-randomised studies	not serious	not serious	not serious	not serious	publication bias strongly suspected[a]	659/33028 (2.0%)	6390/281179 (2.3%)	**RR 0.48** (0.46 to 0.63)	**12 fewer per 1,000** (from 12 fewer to 8 fewer)	⨁◯◯◯ Very low[a]	
**Respiratory failure**												
5	non-randomised studies	not serious	not serious	not serious	not serious	publication bias strongly suspected[b]	36/6174 (0.6%)	956/106669 (0.9%)	**RR 0.37** (0.18 to 0.75)	**6 fewer per 1,000** (from 7 fewer to 2 fewer)	⨁◯◯◯ Very low[b]	
**ICU**												
5	non-randomised studies	not serious	not serious	not serious	not serious	none	5/21065 (0.0%)	28/23785 (0.1%)	**RR 0.33** (0.13 to 0.84)	**1 fewer per 1,000** (from 1 fewer to 0 fewer)	⨁⨁◯◯ Low	
**Mortality**												
12	non-randomised studies	not serious	not serious	not serious	not serious	none	22/32758 (0.1%)	1312/154977 (0.8%)	**RR 0.16** (0.11 to 0.24)	**7 fewer per 1,000** (from 8 fewer to 6 fewer)	⨁⨁◯◯ Low	

CI: confidence interval; **RR:** risk ratio

Explanations

a. Based on reported Funnel plots and Egger's test
b. Based on Egger's Test
