# Supplementary material for: Pharmacological and Adjunctive Management of Non-Hospitalized COVID-19 Patients During the Omicron Era: A Systematic Review and Meta-Analysis
Source: Viruses. 2025 Aug 16;17(8):1128. doi: 10.3390/v17081128 (PMC12390715; doi:10.3390/v17081128)
Supplement: Supplementary file 1 [file viruses-17-01128-s001.zip › Supplementary material S4b.GRADE Remdesivir vs. no treatment.pdf]

Author(s):
Question: Remdesivir compared to no treatment for Hospitalization/Respiratory failure/ICU/Mortality reduction
Setting:
Bibliography:

Certainty assessment							№ of patients		Effect		Certainty	Importance
**№ of studies**	**Study design**	**Risk of bias**	**Inconsistency**	**Indirectness**	**Imprecision**	**Other considerations**	**Remdesivir**	**no treatment**	**Relative (95% CI)**	**Absolute (95% CI)**		
**Hospitalization**												
7	non-randomised studies	not serious	not serious	not serious	not serious	none	60/2248 (2.7%)	329/3932 (8.4%)	**RR 0.30** (0.19 to 0.47)	**59 fewer per 1,000** (from 68 fewer to 44 fewer)	⨁⨁◯◯ Low	
**Respiratory failure**												
3	non-randomised studies	not serious	not serious	not serious	not serious	none	3/409 (0.7%)	64/488 (13.1%)	**RR 0.11** (0.03 to 0.44)	**117 fewer per 1,000** (from 127 fewer to 73 fewer)	⨁⨁◯◯ Low	
**ICU**												
4	non-randomised studies	not serious	not serious	not serious	not serious	none	4/1661 (0.2%)	22/2965 (0.7%)	**RR 0.45** (0.18 to 1.13)	**4 fewer per 1,000** (from 6 fewer to 1 more)	⨁⨁◯◯ Low	
**Mortality**												
7	non-randomised studies	not serious	not serious	not serious	not serious	none	17/2248 (0.8%)	59/3932 (1.5%)	**RR 0.59** (0.35 to 1.01)	**6 fewer per 1,000** (from 10 fewer to 0 fewer)	⨁⨁◯◯ Low	

CI: confidence interval; **RR:** risk ratio
