# Supplementary material for: Pharmacological and Adjunctive Management of Non-Hospitalized COVID-19 Patients During the Omicron Era: A Systematic Review and Meta-Analysis
Source: Viruses. 2025 Aug 16;17(8):1128. doi: 10.3390/v17081128 (PMC12390715; doi:10.3390/v17081128)
Supplement: Supplementary file 1 [file viruses-17-01128-s001.zip › Supplementary material S4c. GRADE Sotrovimab vs. no treatment.pdf]

Author(s):
Question: Sotrovimab compared to no treatment for Hospitalization/Respiratory failure/ICU/Mortality reduction
Setting:
Bibliography:

Certainty assessment							№ of patients		Effect		Certainty	Importance
№ of studies	Study design	Risk of bias	Inconsistency	Indirectness	Imprecision	Other considerations	Sotrovimab	no treatment	Relative (95% CI)	Absolute (95% CI)		
**Hospitalization**												
6	non-randomised studies	not serious	not serious	not serious	not serious	none	222/6567 (3.4%)	22745/545335 (4.2%)	**RR 0.71** (0.54 to 0.93)	**12 fewer per 1,000** (from 19 fewer to 3 fewer)	⨁⨁◯◯ Low	
**Respiratory failure**												
3	non-randomised studies	not serious	not serious	not serious	not serious	none	9/1650 (0.5%)	51/3850 (1.3%)	**RR 0.37** (0.19 to 0.76)	**8 fewer per 1,000** (from 11 fewer to 3 fewer)	⨁⨁◯◯ Low	
**ICU**												
3	non-randomised studies	not serious	not serious	not serious	not serious	none	4/1617 (0.2%)	27/3786 (0.7%)	**RR 0.40** (0.15 to 1.04)	**4 fewer per 1,000** (from 6 fewer to 0 fewer)	⨁⨁◯◯ Low	
**Mortality**												
6	non-randomised studies	not serious	not serious	not serious	not serious	none	10/6567 (0.2%)	2829/545330 (0.5%)	**RR 0.34** (0.19 to 0.61)	**3 fewer per 1,000** (from 4 fewer to 2 fewer)	⨁⨁◯◯ Low	

CI: confidence interval; **RR:** risk ratio
