# Supplementary material for: Pharmacological and Adjunctive Management of Non-Hospitalized COVID-19 Patients During the Omicron Era: A Systematic Review and Meta-Analysis
Source: Viruses. 2025 Aug 16;17(8):1128. doi: 10.3390/v17081128 (PMC12390715; doi:10.3390/v17081128)
Supplement: Supplementary file 1 [file viruses-17-01128-s001.zip › Supplementary material S4d.GRADE Molnupiravir vs. no treatment.pdf]

Author(s):
Question: Molnupiravir compared to no treatment for Hospitalization/Respiratory failure/Mortality reduction
Setting:
Bibliography:

Certainty assessment							№ of patients		Effect		Certainty	Importance
№ of studies	Study design	Risk of bias	Inconsistency	Indirectness	Imprecision	Other considerations	Molnupiravir	no treatment	Relative (95% CI)	Absolute (95% CI)		
**Hospitalization**												
6	non-randomised studies	not serious	not serious	not serious	not serious	none	701/6898 (10.2%)	3426/108570 (3.2%)	**RR 0.80** (0.70 to 0.91)	**6 fewer per 1,000** (from 9 fewer to 3 fewer)	⨁⨁◯◯ Low	
**Respiratory failure**												
4	non-randomised studies	not serious	not serious	not serious	not serious	none	31/6211 (0.5%)	876/106852 (0.8%)	**RR 0.45** (0.27 to 0.77)	**5 fewer per 1,000** (from 6 fewer to 2 fewer)	⨁⨁◯◯ Low	
**Mortality**												
7	non-randomised studies	not serious	not serious	not serious	not serious	publication bias strongly suspected[a]	97/12243 (0.8%)	1254/132000 (0.9%)	**RR 0.64** (0.52 to 0.79)	**3 fewer per 1,000** (from 5 fewer to 2 fewer)	⨁◯◯◯ Very low[a]	

CI: confidence interval; **RR:** risk ratio

Explanations

a. Based on Egger's Test
